# Frequency of Functional Constipation in Lebanese Children: A Cross-Sectional Study Based on Parental Reporting

**DOI:** 10.1155/2024/5183069

**Published:** 2024-08-24

**Authors:** Theresia Tannoury, Jana Assy, Nadine Yazbeck

**Affiliations:** ^1^ Department of Pediatrics and Adolescent Medicine American University of Beirut, Beirut, Lebanon; ^2^ Department of Pediatrics University Hospital of Liege, Liege, Belgium

**Keywords:** children, frequency, functional constipation, Lebanon

## Abstract

**Aim:** To determine the frequency and possible associated dietary and environmental factors of functional constipation (FC) among children in Lebanon followed at a single pediatric health system.

**Method:** A prospective cross-sectional study was conducted in all pediatrics clinics at the American University of Beirut Medical Center (AUBMC). Children aged 2–7 years presenting for a well-child visit were recruited. Data relating to the child's bowel habits and other history items were obtained from parental questionnaires.

**Results:** The mean age of the 172 recruited participants was 4.94 years with 56.4% being males. FC was present in 32.6% of the participants. Although there was no difference in the frequency of FC based on age and gender, the peak frequency of FC was at 5 years. The daily frequency of withholding stools was 64.3%, and 46.6% of the children with FC always experienced straining while stooling for the past 2 months. Decreased physical activity and diet were not significantly associated with FC.

**Conclusion:** The present study shows that 32.6% of children aged 2–7 years in Lebanon suffer from constipation while only 51.7% of the recruited children's physicians inquire about the child's bowel movement during the well check visit. These numbers highlight the need to raise more awareness among pediatricians on the need to screen for constipation during clinic visits as a standard of care practice.

## 1. Introduction

Childhood constipation is one of the most common functional gastrointestinal disorders worldwide and a growing global public health issue [1–5]. The prevalence of functional constipation (FC) ranges from 0.7% (in Europe) to 30% (in Asia) [1] depending on the variability in the various definitions of constipation and the different diagnostic criteria utilized [5].

While the pathophysiology of FC in children remains unclear, it is multifactorial [1] and has been noted in some cases to be present in higher prevalence among patients with certain conditions like celiac disease or cow milk allergy [6]. Moreover, the variability in prevalence depends on several risk factors including the demographic, behavioral, and socioeconomic variables with a peak prevalence during the preschool years [7]. Although constipation occurs in all continents, there is scarce research on the prevalence and frequency data in children from Lebanon [4].

The clinical symptoms of FC range from infrequent bowel evacuation, hard small feces, difficult or painful evacuation of large-diameter stools, and fecal incontinence [4, 8, 9]. Fecal incontinence can be the sole presenting symptom in approximately 10% of children [4, 5, 7, 10] which can be easily missed or misinterpreted by the caregivers [4, 5, 7–11].

When the symptoms of stool-withholding behavior are left unnoticed or not addressed, this may result in fecal incontinence. This behavior will perpetuate as a vicious cycle where stool is withheld to avoid the pain of defecation until eventually, the rectum is overly distended resulting in overflow fecal incontinence [12]. Therefore, early detection and intervention are critical to prevent the development of chronic constipation and its impact on the child's psychological and physical development [4, 5, 7–10].

Although constipation is common and has a set of clear guidelines for diagnosis, there is a difference in the cultural lack of awareness of childhood constipation as well as variability in diagnostic criteria used in each country [5, 8–16]. That said, there have been numerous attempts to standardize the definition of constipation in children via the Iowa, Rome II, Rome III, and Rome IV criteria to facilitate the diagnosis and thus management of constipation in children [16].

To date, there are scarce studies that reflect on the prevalence and frequency of childhood constipation in Lebanon and, in turn, the degree of awareness present in the community. The objective of this study is to determine the frequency of possible associated dietary and environmental factors with the rate of FC among children in Lebanon followed at a single pediatric health system.

## 2. Method

### 2.1. Study Design and Setting

A cross-sectional study was conducted in all pediatrics clinics at the American University of Beirut Medical Center (AUBMC). AUBMC is one of the largest medical center hospitals in Lebanon. Data were collected from June 2018 to June 2019 after obtaining the oral consent from parents of the pediatric population (aged 2–7 years) presenting to different clinics at AUBMC. Parents were approached in the clinics' waiting area discreetly and were given a hard copy of the study forms (Supporting Information (available here)) to fill while waiting.

### 2.2. Study Instruments and Data Collection

#### 2.2.1. Data Collection

The questionnaire used in the study was adopted and modified from a validated questionnaire used by Udoh et al. (Supporting Information) [17]. The questionnaire entailed questions related to the sociodemographic characteristics and exposure to stressful life events in addition to the pediatric gastrointestinal symptoms based on Rome IV criteria and the Modified-Bristol Stool Form Scale.

Before administering, the research team member pilot tested the survey on 10 parents to check clarity and applicability. The questionnaire was administered to parents in an isolated corner at the waiting area in the pediatrics clinics at AUBMC.

### 2.3. Study Sample

#### 2.3.1. Sample Size Determination

Since there is no report on the prevalence of FC among children in Lebanon for the studied age group, the prevalence of 12% obtained by Scarpato et al. [3] for Lebanon among children between the ages of 4 years to 10 years was used to calculate the minimum sample size. A 95% CI, 5% with 1.96 as standard normal deviation and 20% attrition, and a population size of 665,760 for age group 2–7 years as obtained from the United Nations Population Division were used in the calculation. Based on the above, the minimum sample size for the study was 163.

### 2.4. Study Participants

Study participants were gathered consecutively in each clinic with inclusion criteria: 2–7 years of age and generally in a good health condition. Data included into this study was obtained using the adopted questionnaire. Exclusion criteria were patients with known chronic diseases that predispose to constipation as well as patients presenting to the clinic for constipation. The study was eligible for the exemption category from the Institutional Review Board (IRB)/Human Research Protection Program (HRPP) at the American University of Beirut since it involves interview procedures and the human subject can not be identified. The survey responses were all anonymous.

### 2.5. Data Analysis

Data were entered into Excel and analyzed using the Statistical Package for Social Sciences (SPSS) version 25.0 (for Windows IBM, Armonk, NY). Results were expressed as percentages.

Baseline characteristics were summarized as proportions and percentages for categorical variables. Continuous data were presented as median ± SD. Categorical data were expressed as numbers (percentage). The *χ*^2^ test was used to test the association between categorical variables and FC. A *p* value of < 0.05 was deemed to be statistically significant. Correlation between constipation and bowel habits among the constipated population was used with a *p* value of < 0.01 deemed to be statistically significant.

## 3. Results

### 3.1. Sample Characteristics and Frequency of FC

Two hundred parents were approached for participation; of these, 28 declined. A total of 172 questionnaires were filled and included in the final analysis.  Table 1 shows the distribution of subjects according to sociodemographic characteristics and frequency of constipation in each category. There was no significant association between FC and any of the four variables including age, gender, number of households, and number of siblings. The mean age of the children included in the study was 4.94 years (SD 1.35 years, range 3–7 years) with 97 (56.4%) being males.

**Figure 1:**
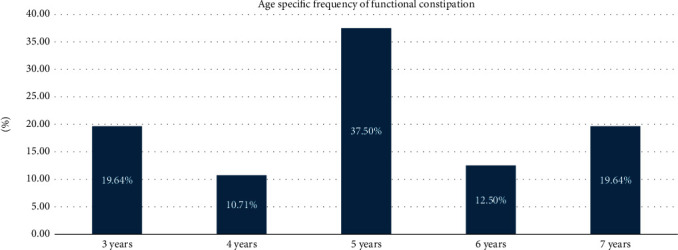
Age-specific frequency of functional constipation (FC) among the studied population.

FC was present in 56 (32.6%) of the participants. The mean age of children with FC was 5.02 years (SD 1.355 years, range 3–7 years) and 4.91 years (SD 1.351 years, range 3–7 years) for those without the condition. The peak age frequency was at 5 years while the lowest frequency was at 4 years (Figure 1).

**Figure 2:**
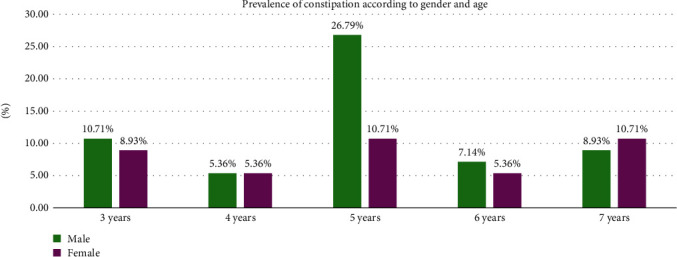
Frequency of function constipation according to gender and age.

The peak frequency of FC in males was 26.79% at 5 years of age while the peak frequency in females was 10.71% at 5 and 7 years of age (Figure 2).

### 3.2. Stressful Factors and Constipation

Table 2 depicts the association between constipation and exposure to stressful life events. None of the stressful events evaluated in this study was significantly associated with FC (Table 2).

### 3.3. Constipation History and Treatment

Out of the 172 children, 78 (45.3%) had a history of constipation with 83.3% being identified by parents and 16.7% diagnosed by pediatricians. These past constipation episodes were mainly managed by pediatricians (71.8%), followed by parents (26.9%), and pharmacists (1.3%). The treatment of past constipation was equally controlled by either medication (50%) or dietary change (50%). It is worth noting that history of constipation, as perceived by parents, had a significant correlation with the status of constipation with a *p* value < 0.001 (Table 3).

### 3.4. Eating/Drinking Habits and Physical Activity

Table 4 depicts the association between constipation and eating/drinking habits and physical activity. None of the eating/drinking habits and physical activity evaluated in this study was significantly associated with FC (Table 4).

### 3.5. Bowel Habits of Children With Constipation

Bowel habits of children with constipation are depicted in Table 5. Withholding behavior was present in 64.3% of the children with constipation. Defecation two times a week or less was found in 10.7% of the children. Presence of hard stools was seen in 25.0% (Table 5).

The most common bowel-related symptoms associated with constipation were pain while stooling in the last 2 months accounting for 67.9% and always having to strain (push hard) while stooling in the last 2 months accounting for 46.6%.

### 3.6. Pediatrician Visit and Bowel Movement Screening

The frequency of the pediatrician visits had no association with FC (Table 6). It is worth noting that 51.7% of pediatricians ask about the child's bowel movements, followed by 15.7% sometimes asking about the child's bowel movements, and 32.6% never asking about the child's bowel movements.

## 4. Discussion

To the best of our knowledge, to date, there are no data in the literature that report the incidence of FC and study its relationship with the sociodemographic factors and stressful events among the pediatric population aged 2–7 years in Lebanon based on Rome IV criteria and the Modified-Bristol Stool Form. This study shows an overall frequency of FC to be 32.6% in the studied population.

The frequency rate of constipation in Lebanon is slightly higher than most of the studies from the West but is similar to the prevalence rate of constipation reported by Oswari et al. [19] in Taiwan (32.2%) [1, 17, 19]. Our finding was higher than that reported by Scarpato et al. [3] who reported FC among 11.4% of children aged 4–10 years in Lebanon and 14.4% of children aged 4–10 years in Jordan. Although there is no specified reason regarding such variability in the rate, the difference can be attributed to the sample selection; difference in the data collection instrument; cultural differences in the interpretation of bowel habits and other GI symptoms; differences in the behavioral patterns; difference in genetic potential in developing constipation in the different studied populations; and the instability in the health, social, economic, and political determinants of the region which are considered stressful events that may increase the risk of developing FC [10, 17, 19].

Although there was no difference in the frequency of FC based on age and gender, the peak frequency of FC was at 5 years (37.5%) while the lowest frequency was at 4 years (10.7%). In males, the peak frequency was at 5 years of age (26.79%) while the peak frequency in females was at 5 and 7 years of age (10.71%). Unlike the literature, the highest frequency of FC in our studied population was among those aged 5 years rather than children older than 6 years. This difference can be explained by the fact that in Lebanon, children aged 3 years become school-aged in comparison to the other countries where children start attending school at the age of 6 years [19, 20].

The high level of psychological stress caused by the school environment is considered a stressful event that predisposes and predicts FC development among school-aged children [19, 20].

The study showed no overall difference in the frequency of FC based on gender. However, FC frequency in males was higher in all studied ages from 3 to 6 years, but at the age of 7 years, the frequency of FC among the females became higher than that in males. There is no conclusive explanation regarding the predominance of gender in constipation with studies either exhibiting no difference in FC between males and females such as those conducted by Rajindrajith et al. [21] and Lu et al. [22] or exhibiting a predominance among males such as that conducted by Lewis et al. [7] and other exhibiting predominance among females especially as they get older in age such as those conducted by Wu et al. [16], Chu et al. [20], and Oswari et al. [19]. The higher prevalence of FC among females, especially as they get older, is attributed to hormonal factors [17, 19, 20].

Unlike other studies that associate FC with socioeconomic status, stressful life events, or the psychological strain caused by the negative perceptions of school toilets leading to the adoption of unhealthy toilet habits during school time [7, 17, 19–23], in this study, none of the social background factors nor the stressful life events assessed were significantly associated with FC. The reason for such insignificant outcomes may be explained by the small sample size as well as the factors of social indicators and stressful events evaluated in this study.

In this study, decreased physical activity was not significantly associated with FC. It is worth noting that while Macêdo et al. [24] found no association between FC and physical inactivity, Leung and Hon [25] and Vriesman et al. [2] reported that decreased physical activity was considered an important predictor and risk factor for constipation in children.

While Leung and Hon [25] reported that high dietary fiber intake was significantly associated with a lower prevalence of constipation, Tappin et al. [26] reported that increasing the fiber intake did not effectively treat constipation. In this study, dietary practices that entail the increase in fiber intake (fruits and vegetables) were not significantly associated with decreased constipation prevalence. That said, it is worth noting that the increase of FC prevalence among the school-aged population might depend more on the change in dietary habits between the home environment and the school lunches which are also mediated by a decrease in fluid intake and low fiber diet which leads to the development of hard stools as well as the psychological strain that the child faces during this phase [27].

While parents (83.3%) diagnosed the constipation among the children in the study, the rate of consulting a medical professional (physician and pharmacist) to manage constipation was considered desirable with 71.8% of the cases being managed by the pediatrician and 1.3% being managed by a pharmacist. This rate was higher than that reported by Inan et al. [23]. The constipation treatment mode was equally distributed between medication treatment (50%) and diet regiment (50%) which differs from the treatment mode described by Inan et al. [23] who reported that most of the constipated children who had a medical consultation for constipation received only drug therapy.

It is worth noting that the frequency of the doctors' visit had a negative/inverse relation with the current status of constipation and was found to be significant at a *p* value = 0.001.

Pain while stooling was higher than that reported by Chogle and Saps [28] (54.9%) and Rajindrajith et al. [21] (55%) and was lower than that reported by Oswari et al. [19] which reached 91% among the studied children [29]. In the study, 46.6% of the children with FC always experienced straining while stooling for the past 2 months. Straining was lower than that reported by Rajindrajith et al. [21] (71.6%). The daily frequency of stained/soiling stools was 14.3% in our study which is significantly lower than that reported by Dehghani et al. [29] which reached 40.8%. The daily frequency of withholding stools was 64.3% in our study which is similar to that reported by Oswari et al. [19] (68.3%) and lower than that reported by Mutyala, Sanders, and Bates [27] reaching 92.0%.

Only 51.7% of the physicians in the study inquired about the child's bowel movement during the well check visit. Pediatricians ought to address bowel movement screening during clinic visits as a standard of care practice in order to diagnose and offer management early on for FC.

Our study had several limitations: the main one being the collection of data from a single tertiary care center in Beirut which would constitute a selection bias leading to higher frequency. Another limitation is the use of a questionnaire which may be subject to information bias, including recall bias. The third limitation would be the noninclusion of major life events that may be considered as stressors or risk factors such as but not limited to suspension from school, frequent punishment in school, separation from best friend, and being bullied at school which are known to be associated with FC. As well as the lack of inclusion of some factors such as fluid intake and use of medications and supplements in the questionnaire, that may have an impact on FC development.

## 5. Conclusion

The present study shows that 32.6% of children aged 2–7 years in Lebanon suffer from constipation. This growing health problem among children requires the attention of pediatricians, primary care physicians, and policy makers in Lebanon. It is also worth stating that around 32.6% of the recruited children's physicians never inquired about the child's bowel movements, and 15.7% sometimes inquired about the child's bowel movements during the well check visit. These numbers emphasize the need to raise more awareness among pediatricians regarding screening for bowel movement during clinic visits as a standard of care practice. Adopting a constipation-screening tool based on the latest updated guidelines while adjusting it according to each service/provider's practice could ensure proper detection of the signs and symptoms of constipation during well-child visits.

## Figures and Tables

**Table 1:** Rome IV criteria to diagnose functional constipation (FC) [18].

**Children aged 4 years and younger**	**Children aged 4 years and older**
A child is defined as having functional constipation if he/she has 2 or more of the following for at least 1 month: • ≤ 2 defecations per week • History of excessive stool retention • History of painful or hard bowel movements • Presence of a large fecal mass in the rectum • History of large-diameter stools In children who are toilet trained, the below additional criteria may be used: • At least 1 episode per week of incontinence after the acquisition of toileting skills	A child is defined as having functional constipation if he/she has 2 or more of the following for at least 1 month: • ≤ 2 defecations in the toilet per week • At least 1 episode of fecal incontinence per week • History of retentive posturing or excessive volitional stool retention • History of painful or hard bowel movements • Presence of a large fecal mass in the rectum • History of large-diameter stools (may block the toilet)

**Table 2:** Distribution of respondents according to exposure risk factors and stressful life events.

**Variable**	**Distribution of subjects**		**p** ** value**
	**Control** **N** = 116	**Children with constipation** **N** = 56	
Chronic medical conditions			
Yes	12 (10.3%)	5 (8.9%)	0.771
No	104 (89.7%)	51 (91.1%)	
Birth order			
1	61 (52.6%)	32 (57.1%)	0.756
2	36 (31.0%)	15 (26.8%)	
3	13 (11.2%)	5 (8.9%)	
4	4 (3.4%)	4 (7.1%)	
5	1 (0.9%)	0 (0.0%)	
6	1 (0.9%)	0 (0.0%)	
Change house/school in the past year			
No	93 (80.2%)	37 (66.1%)	0.050
School	18 (15.5%)	10 (17.9%)	
House	4 (3.4%)	6 (10.7%)	
School and house	1 (0.9%)	3 (5.4%)	
Started daycare or school			
No	9 (7.8%)	4 (7.1%)	0.359
Daycare	8 (6.9%)	1 (1.8%)	
School	99 (85.3%)	51 (91.1%)	
Toilet trained			
Yes	112 (96.6%)	54 (96.4%)	0.967
In process	0	0	
No	4 (3.4%)	2 (3.6%)	
Uses the daycare/school toilet			
Yes	80 (69.0%)	33 (58.9%)	0.223
No	21 (18.1%)	18 (32.1%)	
Not sure/not applicable	15 (13.0%)	5 (9.0%)	

**Table 3:** Functional constipation history and treatment mode.

**Constipation history**	**Category**	**n** ** (percent)**
FC diagnosis^a^	Parents	65 (83.3%)
	Pediatrician	13 (16.7%)

FC management	Parents	21 (26.9%)
	Pediatrician	56 (71.8%)
	Pharmacist	1 (1.3%)

FC treatment	Medication	39 (50.0%)
	Diet	39 (50.0%)

^a^ *χ* ^2^ < 0.001.

**Table 4:** Distribution of the respondents according to their eating/drinking habits and physical activity.

**Variable**	**Distribution of subjects**	
	**Control** **N** = 116	**Children with constipation** **N** = 56
Physical activity^a^		
Two times per week	82 (70.7%)	40 (71.4%)
Once per week	22 (19.0%)	10 (17.9%)
Occasionally	10 (8.6%)	4 (7.1%)
Never	2 (1.7%)	2 (3.6%)
Frequency of drinking milk a day^b^		
More or equal to 3 cups per day	10 (8.6%)	3 (5.4%)
One to 2 cups per day	49 (42.2%)	34 (60.7%)
Does not drink milk	57 (49.1%)	19 (33.9%)
Frequency of fiber intake (fruits and vegetables) per day^c^		
Three or more	42 (36.2%)	15 (26.6%)
1 to 2 fruit/vegetable	62 (53.4%)	31 (55.4%)
Does not eat fruits/vegetables regularly	12 (10.3%)	10 (17.9%)

^a^ *χ* ^2^ = 0.877.

^b^ *χ* ^2^ = 0.075.

^c^ *χ* ^2^ = 0.259.

**Table 5:** Bowel habits of children with functional constipation.

**Bowel habit**	**Category**	**n** ** (percent)**
Frequency of stools in the last 2 months	Two times a week or less	6 (10.7%)
	Three to 6 times a week	16 (28.6%)
	One or more a day	34 (60.7%)

Bristol stool type	Type 1	9 (16.1%)
	Type 2	10 (17.9%)
	Type 3	22 (39.3%)
	Type 4	15 (26.8%)
	Type 5	0
	Type 6	0
	Type 7	0

Period of hard stools	Not applicable	29 (51.8%)
	Less than 1 month	9 (16.1%)
	2 months	4 (7.1%)
	Three or more months	14 (25.0%)

Pain while stooling in the last 2 months	No	18 (32.1%)
	Yes	38 (67.9%)

Strain (push hard) while stooling in the last 2 months	Never	5 (8.9%)
	Once in a while	4 (7.1%)
	Sometimes	15 (26.8%)
	Most of the time	4 (7.1%)
	Always	26 (46.4%)

Rushing to bathroom	Never	12 (21.4%)
	Sometimes	9 (16.1%)
	Most of the time	6 (3.5%)
	Always	33 (19.2%)

Stained/soiled underwear in the last 2 months	Never	32 (57.1%)
	Less than once a month	4 (7.1%)
	1–3 times a month	5 (8.9%)
	Once a week	5 (8.9%)
	Several times a week	2 (3.6%)
	Everyday	8 (14.3%)

Big stools that clogged the toilet in the last 2 months	No	44 (78.6%)
	Yes	12 (21.4%)

Withhold stools in the last 2 months	Never	10 (17.9%)
	1–3 times a month	2 (3.6%)
	Once a week	1 (1.8%)
	Several times a week	7 (12.5%)
	Everyday	36 (64.3%)

**Table 6:** Pediatrician visit frequency and bowel movement screening.

**Variable**	**Distribution of subjects**		**Total**
	**Control** **N** = 116	**Children with constipation** **N** = 56	
Frequency of pediatrician visits^a^			
Very often	45 (38.0%)	19 (33.9%)	64 (37.2%)
For vaccination only	29 (25.0%)	20 (35.7%)	49 (28.5%)
Occasionally	42 (36.2%)	17 (30.4%)	59 (34.3%)
Screening for constipation^b^			
Always	64 (55.2%)	25 (44.6%)	89 (51.7%)
Sometimes	16 (13.8%)	11 (19.6)	27 (15.7%)
Never	36 (31.0%)	20 (35.7%)	56 (32.6%)

^a^ *χ* ^2^ = 0.8389.

^b^ *χ* ^2^ = 0.343.

## Data Availability

The raw data supporting the conclusions of this manuscript will be made available by the authors, without undue reservation, to any qualified researcher.

## Nomenclature

AUBMC: American University of Beirut Medical Center
FC: functional constipation
HRPP: Human Research Protection Program
IRB: Institutional Review Board
SD: standard deviation
SPSS: Statistical Package for Social Sciences
