# Supplementary material for: Frequency of Functional Constipation in Lebanese Children: A Cross-Sectional Study Based on Parental Reporting
Source: Int J Pediatr. 2024 Aug 24;2024:5183069. doi: 10.1155/2024/5183069 (PMC11366055; doi:10.1155/2024/5183069)
Supplement: Supporting Information — Additional supporting information can be found online in the Supporting Information section. [file 5183069.f1.docx]

**
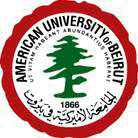
**

Fellowship and Residency Research Program (FRRP) Faculty of Medicine

American University of Beirut

Beirut, Lebanon

**Appendix A**

**Data Collection Sheet**

1. **Gender □** Male □ Female
2. **Height (cm): ____**
3. **Weight (kg): ____**
4. **Date of birth: __/__/____ Age :** ____________
5. **Today’s date: __/__/____**
6. **Does your child have any chronic medical conditions?**

**□** No □ Diabetes □ Heart disease □ neurologic

□ Other: ____________

1. **Number of household members: ______**
2. **Number of siblings: ______**
3. **Rank in the family: ______**
4. **Did your child ever have constipation?**

□ yes □ no

**11. If you answer to question number 10 as yes please answer the following 3 questions:**

**11.1 How was your child diagnosed with constipation?**

□ by his pediatrician □ by yourself □ other ____________

**11.2 Who managed your child’s constipation?**

□ yourself □ The pharmacist □ The pediatrician □ other ____________

**11.3 How was your child managed for constipation?**

□ Diet □ Medication (name: ) □ other ____________

**12. How often do you visit your pediatrician?**

□ Very often □ For vaccination only □ occasionally

**13. Does your pediatrician ask you about your child’s bowel movements pattern?**

□ Always □ sometimes □ never

**14. How often is your child involved in a physical activity?**

□ 2 times/ week □ once per week □ occasionally □ never

**15. How much milk does your child drink a day?**

□ doesn’t drink milk on daily basis □ one to 2 cups per day □ more equal to 3 cups per day

**16. How much does your child eat fibers (fruits and vegetables) per day?**

□ doesn’t eat fruits and vegetables regularly □ 1 to 2 fruit/vegetable □ 3 or more

**17. In the past year did your child change:**

School house

**18.Does your child go to daycare or school?**

□ No □ daycare □ school

**19. Is your child toilet trained?**

□ Yes □ in the process □ no, not yet

**20. Does your child use the daycare or the school’s toilet?**

□ yes □ no □ I am not sure

**21. In the last 2 months, how often did your child usually have stools?**

| 1. 2 times a week or less often |  |
| --- | --- |
| 2. 3 to 6 times a week |  |
| 3. Once or more a day |  |

**22. In the last 2 months, what was your child’s stool usually like?**

**
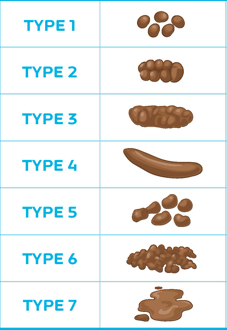
**

**23. If your child stools are usually hard, for how long have they been hard?**

| 0. Less than 1 month |  |
| --- | --- |
| 1. 1 month |  |
| 2. 2 months |  |
| 4. 3 or more months |  |

**24. In the last 2 months, did your child complain of pain while stooling?**

0. No

1. Yes

**25. In the last 2 months, did your child have to strain (push hard) to make a stool come out?**

0. Never 1. Once in a while 2. Sometimes

3. Most of the time 4. Always

**26. If your child is toilet trained please answer the following 2 questions.**

**Did he/she have to rush to the bathroom to stool?**

0. Never 1. Once in a while 2. Sometimes

3. Most of the time 4. Always

**27. In the last 2 months, how often have you seen that your child’s underwear is stained or soiled with stool?**

| 0. Never. |  |
| --- | --- |
| 1. Less than once a month |  |
| 2. 1 – 3 times a month |  |
| 3. Once a week. |  |
| 4. Several times a week |  |
| 5. Every day |  |

**28. In the last 2 months, did your child have a stool that was so big that it clogged the toilet?**

0. No

1. Yes

**29.** Some children hold in their stool even when there is a toilet they could use. They may do this by stiffening their bodies or crossing their legs. In the last 2 months, while at home, how often have you seen this behaviors?

| 0. Never. |  |  |
| --- | --- | --- |
| 1. 1 – 3 times a month |  |  |
| 2. Once a week. |  |  |
| 4. Several times a week |  |  |
| 5. Every day |  |  |
